# Draft Genome Sequence of *Lactobacillus plantarum* 2025

**DOI:** 10.1128/genomeA.01532-15

**Published:** 2016-01-07

**Authors:** Andrey V. Karlyshev, Valentin C. Khlebnikov, Igor V. Kosarev, Vyacheslav M. Abramov

**Affiliations:** a School of Life Sciences, Pharmacy and Chemistry, Faculty of Science, Engineering and Computing, Kingston University, Kingston upon Thames, United Kingdom; b Institute of Immunological Engineering, Lyubuchany, Moscow Region, Russia

## Abstract

A draft genome sequence of *Lactobacillus plantarum* 2025 was derived using Ion Torrent sequencing technology. The total size of the assembly (3.33 Mb) was in agreement with the genome sizes of other strains of this species. The data will assist in revealing the genes responsible for the specific properties of this strain.

## GENOME ANNOUNCEMENT

Although the beneficial effects of probiotics on human health are well known, the molecular mechanisms of their action are not completely clear (reviewed in Behnsen et al. [1]). The properties of a species may vary depending on the strain. For example, a comparative analysis of 20 strains of *Lactobacillus plantarum* revealed significant variation in their resistance to acidic conditions and the ability to attach to Caco-2 cells (2).

Our investigation of the vaginal bacterial flora resulted in the isolation of 120 strains of *L. plantarum*. Thirty-five of these stains were resistant to gastrointestinal stress conditions. Ten of these isolates were found to be highly adhesive to human tissue cultures. Only one of these strains, *L. plantarum* 2025, revealed symbiotic and synergistic effects upon application with other probiotic strains. This strain, deposited at the All-Russian Collection of Microorganisms at the G. K. Skryabin Institute of Biochemistry and Physiology of Microorganisms (Russian Academy of Sciences, Pushchino, Russia) under registration number VKM V-2731D, presents significant interest for the development of antibacterials based on probiotics.

The sequencing reads were generated by the Ion Torrent PGM using a 314 Chip version 2 and a 400-bp sequencing kit. Initial assembly of the sequences with the Torrent SPAdes plugin and Genomics Workbench software (GWB) produced contigs not exceeding 71 kb. GWB reads mapping on the genome of *L. plantarum* strain B21 (3) showing the highest similarity allowed the generation of consensus sequences with up to 140 kb. The consensus sequences were combined with Torrent SPAdes and GWB assemblies using the CISA contig integrator (4) and further assembled using GWB, resulting in 164 contigs >1 kb and up to 187.6 kb, with a total size of 3,334,257 bp (28.02× coverage). Both the size and G+C content (44.4%) were in good agreement with these figures for the published complete genome sequences of other strains of this species (3.20 to 3.36 Mb and 44.3 to 44.7%).

The NCBI GenBank genome annotation pipeline allowed the identification of 2,819 protein-coding genes, 47 of which encode putative cell surface/adhesion proteins (up to 1,135 amino acids in size). The genes responsible for bacteriocin production and immunity were found. Similar to other strains of the species, *L. plantarum* 2025 contains the genes responsible for capsular polysaccharide (CPS) biosynthesis.

Using RAST genome annotation server (5), 3,183 protein-coding genes were found. One remarkable feature is the number of genes (368) devoted to carbohydrate metabolism. A gene encoding a fibronectin-binding adhesin was also detected. Among the genes from B21 not present in 2025 were those encoding various hypothetical and membrane proteins, bacteriocin immunity proteins, transposases, cellulose synthase, and restriction-modification proteins. Large (>20 kb) gene clusters containing the genes encoding nonribosomal peptide synthetases NpsA and NpsB, sugar biosynthesis and transport proteins, and prophages were also missing. Among the genes present in both strains but showing significant sequence variation were those encoding MarR (a transcription regulator) and the cell shape-determining protein MreB.

The draft genome sequence of *L. plantarum* 2025 determined in this study is essential for the identification of specific genetic features of this strain and for understanding the mechanisms of its probiotic activity.

### Nucleotide sequence accession numbers.

This whole-genome shotgun project has been deposited at DDBJ/EMBL/GenBank under the accession number AVFJ00000000. The version described in this paper is version AVFJ02000000.
